# *Wealth*-related inequalities of women’s knowledge of cervical cancer screening and service utilisation in 18 resource-constrained countries: evidence from a pooled decomposition analysis

**DOI:** 10.1186/s12939-020-01159-7

**Published:** 2020-03-26

**Authors:** Rashidul Alam Mahumud, Syed Afroz Keramat, Gail M Ormsby, Marufa Sultana, Lal B. Rawal, Khorshed Alam, Jeff Gow, Andre M. N. Renzaho

**Affiliations:** 1 School of Social Sciences, Western Sydney University, Penrith-2751, New South Wales Australia; 2 Translational Health Research Institute (THRI), Western Sydney University, Sydney, New South Wales Australia; 3 Health Economics Research, Health Systems and Population Studies Division, International Centre for Diarrhoeal Disease Research, Bangladesh (icddr,b), Dhaka, Dhaka, 1212 Bangladesh; 4 Health Economics and Policy Research, Centre for Health, Informatics and Economic Research, University of Southern Queensland, Toowoomba, Queensland 4350 Australia; 5 School of Commerce, University of Southern Queensland, Toowoomba, QLD 4350 Australia; 6 Economics Discipline, Social Science School, Khulna University, Khulna, 9208 Bangladesh; 7 Professional Studies, Faculty of Business, Education, Law and Arts, University of southern Queensland, Toowoomba, QLD 4350 Australia; 8 Nutrition and Clinical Services Division, International Centre for Diarrheal Disease Research, Bangladesh (icddr,b), Dhaka-1212, Bangladesh; 9 Deakin Health Economics, School of Health and Social Development, Deakin University, Geelong, Victoria Australia; 10 School of Health Medical and Allied Sciences, CQUniversity Sydney, Sydney, New South Wales Australia; 11 School of Accounting, Economics and Finance, University of KwaZulu-Natal, Durban, 4000 South Africa

**Keywords:** Cervical cancer screening services, Decomposition analyses, Resource-constrained countries, Knowledge, Utilisation

## Abstract

**Introduction:**

Resource-constrained countries (RCCs) have the highest burden of cervical cancer (CC) in the world. Nonetheless, although CC can be prevented through screening for precancerous lesions, only a small proportion of women utilise screening services in RCCs. The objective of this study was to examine the magnitude of inequalities of women’s knowledge and utilisation of cervical cancer screening (CCS) services in RCCs.

**Methods:**

A total of 1,802,413 sample observations from 18 RCC’s latest national-level Demographic and Health Surveys (2008 to 2017–18) were analysed to assess wealth-related inequalities in terms of women’s knowledge and utilisation of CCS services. Regression-based decomposition analyses were applied in order to compute the contribution to the inequality disparities of the explanatory variables for women’s knowledge and utilisation of CCS services.

**Results:**

Overall, approximately 37% of women had knowledge regarding CCS services, of which, 25% belonged to the poorest quintile and approximately 49% from the richest. Twenty-nine percent of women utilised CCS services, ranging from 11% in Tajikistan, 15% in Cote d’Ivoire, 17% in Tanzania, 19% in Zimbabwe and 20% in Kenya to 96% in Colombia. Decomposition analyses determined that factors that reduced inequalities in women’s knowledge of CCS services were male-headed households (− 2.24%; 95% CI: − 3.10%, − 1.59%; *P < 0.01*), currently experiencing amenorrhea (− 1.37%; 95% CI: − 2.37%, − 1.05%; *P < 0.05*), having no problems accessing medical assistance (− 10.00%; 95% CI: − 12.65%, − 4.89%; *P < 0.05*), being insured (− 6.94%; 95% CI: − 9.58%, − 4.29%; *P < 0.01*) and having an urban place of residence (− 9.76%; 95% CI: − 12.59%, − 5.69%; *P < 0.01*). Similarly, factors that diminished inequality in the utilisation of CCS services were being married (− 8.23%;95% CI: − 12.46%, − 5.80%; *P < 0.01*), being unemployed (− 14.16%; 95% CI: − 19.23%, − 8.47%; *P < 0.01*) and living in urban communities (− 9.76%; 95% CI: − 15.62%, − 5.80%; *P < 0.01*).

**Conclusions:**

Women’s knowledge and utilisation of CCS services in RCCs are unequally distributed. Significant inequalities were identified among socioeconomically deprived women in the majority of countries. There is an urgent need for culturally appropriate community-based awareness and access programs to improve the uptake of CCS services in RCCs.

## Background

Cervical cancer (CC) is the fourth leading cancer in women worldwide (570,000 new cases, accounting for 6.6% of all female cancers in 2018) and the eighth-most important cancer overall (contributing 3.3% of the total number of new cases diagnosed in 2018) [1, 2]. In 2018, there were 311,365 estimated deaths from CC worldwide, accounting for 7.5% of all cancer deaths in females, with approximately 90% of deaths occurring in resource-constrained countries (RCCs) [1–4], particularly African ones [5–7]. Infection with the human papillomavirus (HPV) is a key cause of CC and is associated with other anogenital (vulvar, vaginal, penile and anal) cancers [8, 9] as well as head and neck cancers [10]. The burden of CC is a growing public health concern in terms of high incidence and mortality rates worldwide, especially in RCCs [11]. However, the incidence and burden of CC have been drastically reduced in high-income countries in recent decades owing to women accessing CC screening services regularly [12]. Unfortunately, CC still remains the most common cancer among women in RCCs with high rates of associated mortality [12–15].

The high burden of CC in terms of incidence and mortality rates across the world could be decreased (by between one-third and one-half, respectively) with comprehensive primary prevention programs that incorporate early vaccination, early diagnosis, effective screening, adequate referral, and advanced treatment procedures [16–19]. Prevention mechanisms have become more pronounced in the developed world over the past couple of decades, and CC incidence rates have fallen there, largely because of primary prevention programs [19]. During the same period, however, rates in most developing countries have risen or remained unchanged, often based on limited access to health services, lack of awareness and absence of screening and treatment programmes [17, 20–24]. However, screening services are generally the most acceptable prevention strategy, detecting precancerous changes before they progress to the invasive cancer stage [25]. There is significant variation in women’s knowledge about CC and screening services available across countries. Whereas in high-income countries, knowledge about CC and related national screening programs have played a significant role in reducing the burden [22], the level of women’s knowledge about CC and related screening services remains a considerable challenge in RCCs.

The relationship between health care utilisation and socio-demographic characteristics has been widely addressed in the literature [26–30]. Low levels of knowledge and uptake of CC screening services are linked to low socio-economic status (SES) [23, 27, 31], poverty and poor economic development at the country level, and inadequate health services, such as limited health facilities, unaffordability of services, poor quality of cytology services and lack of culturally appropriate and acceptable screening methods [3, 8, 20–22, 26, 32–35].

The utilisation of CC screening services and the burden of CC is disproportionately distributed among poor women globally [36]. For example, just 19% of women utilised CC screening services in RCCs, whereas this figure was over 60% in high-income countries [22]. Further, the utilisation of CC screening services in RCCs ranged from 1.1% in Bangladesh to 57.6% in the Republic of Congo [37]. Substantial heterogeneity in the utilisation of CC screening services was also observed [36], with women from low and middle socioeconomic households receiving 43 and 33% less cervical cancer screening services, respectively than their wealthiest counterparts [37]. Although it is well-established that having an appropriate level of knowledge, awareness, vaccination and regular screening are the most effective ways for preventing CC [28, 35, 38, 39], few studies have attempted to assess the impact of wealth-related inequalities on women’s knowledge of CC and the uptake of CC screening in the context of RCCs.

### Theoretical foundation

This work adopted the socio-ecological model (SEM) as the basis to explain individuals’ health behaviours [40, 41], such as women’s knowledge as well as CC screening participation, within the context of their environments. The SEM facilitates the exploration of the ecological niche (intrapersonal, interpersonal, organisational, community and policy levels) [42]. This framework is important because it allows for the investigation of all salient factors that are essential in policy formulation, which seeks to improve the knowledge and utilisation of CC screening services. At both the individual and micro levels factors like demographic characteristics and SES are considered. The interpersonal level factors include family influences, such as satisfaction in relationships and social support (e.g., support from a spouse and other family members along with relationship power and equity (gender equity)). In terms of the community-level factors, cultural and gender norms, and for the institutional/health systems, factors such as confidence in health care service providers, health insurance coverage and access to health facilities, are considered. Regional wealth inequalities, place of residence and ethnicity are categorised under structural factors, which are driven by the prevailing socio-cultural systems within a country. Even though different levels were distinguished, they were highly interactive because the structural factors function only with the cooperation of individual, interpersonal and institutional factors [43].

## Methods

### The aim of the study

The aim of this study was to examine the inequalities of women’s knowledge and utilisation of CC screening services in 18 RCCs. The point of departure of this study was to hypothesis that knowledge and screening practices of CC among women in RCCs are intricately linked to wealth. This study is the first of its kind that examines the impact of wealth on inequalities of CC screening knowledge and screening in economically poor countries. To achieve the research objective, the following three research questions (RQ) were posited:

RQ 1: What is the level of women’s knowledge about CC services and the level of utilisation in RCCs?

RQ 2: What are the potential factors associated with increased women’s knowledge of CC screening services and their utilisation?

RQ 3: What is the magnitude of wealth inequalities in terms of women’s knowledge about CC screening services and utilisation of screening services in RCCs?

### Study design and settings

**Fig. 1:**
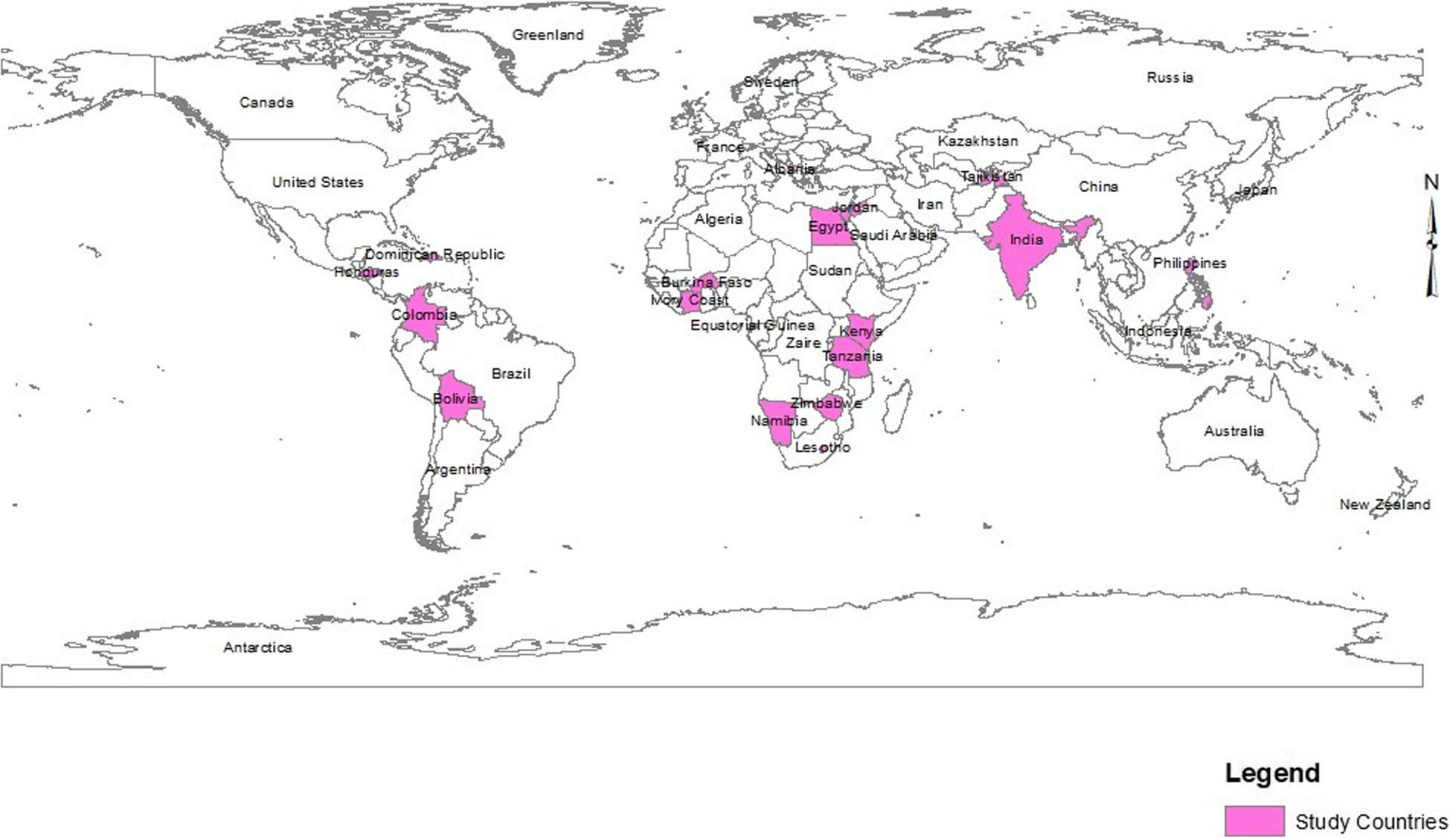
Mapping of the study settings across geographical distribution

This study used data from the Demographic and Health Survey (DHS) conducted across the selected RCCs. As per the study objective(s), only the latest DHS conducted in 18 RCCs were utilised [44]. The DHS is a long-standing worldwide cross-sectional household survey performed in 90 developing countries [44]. Data collection is standardised but the explored health issues vary by country. Hence, data on CC are only available for 18 RCCs. Data captured by the DHS include information on various health indicators related to maternal and child health, maternal and child mortality, fertility, family planning, nutrition, and knowledge and awareness of health, health services and health care utilisation but they vary across countries based on important local health issues. The present study was restricted in 18 resource-constrained countries (RCCs), hence, data on cervical cancer-related information are only available in these countries (Fig. 1). The DHS program collects information on knowledge, awareness and utilisation of CC screening among women from 18 resource-constrained countries only (Fig. 1): Albania (2017–18), Bolivia (2008), Burkina Faso (2010), Colombia (2015–16), Cote d’Ivoire (2011–12), Dominican Republic (2013), Egypt (2015), Equatorial Guinea (2014–15), Honduras (2011–12), India (2015–16), Jordan (2012), Kenya (2014), Lesotho (2014), Namibia (2013), Philippines (2013), Tajikistan (2012), Tanzania (2011–12) and Zimbabwe (2015) (Fig. 1) [44].

The study adopted the World Bank’s definition of resource-constrained countries (RCC), a term used to refer to all countries economically classified as low- or middle-income [45]. The RCCs are typically attributed by a lack of funds to cover health care costs, on individual or societal perspectives, which leads to limited accessibility, affroadibility, accountability and availability of healthcare services in terms of limited infrastructure, poor health systems and delivery mechanisms, and trained personnel [46–48]. Indeed, for weak health care systems, it is plausible that effects beyond women cancer may be realised and may extend to cancer more generally or to women’s health. In addition, LRCs often lack the necessary infrastructure to ensure high-quality cancer screening services and subsequent follow-up care [48]. For example, RCCs often do not have the necessary infrastructure required for ensuring high-quality cancer screening services and associated follow-up care; which in turn may be compromised by the lack of a consistent supply of both electricity, x-ray films, and technicians (engineers, technicians, and radiologists) [46].

### Sampling

A stratified two-stage cluster sampling is used in the most DHS surveys [49]. In the *first stage*, *primary sampling units* (PSUs) are selected from the main DHS sampling framework with probability proportional to a size measure; in the *second stage*, a fixed number of households (or residential dwellings) are selected from a list of households obtained in an updating operation in the selected PSUs using systematic random sampling. A PSU is usually a geographically constructed area, or a part of an area, called an enumeration area (EA), containing a number of households, created from the most recent population census. For simplicity, the DHS surveys captures two-stage surveys: the *first stage* is a systematic sampling with probability proportional to the EA size; the second stage is a systematic sampling of equal probability and fixed size across the EAs. This sampling procedure is usually more precise than simple random sampling at both stages. The detailed sample size calculation procedures are reported elsewhere [49], which depends on a function of the *cost ratio* and the *intracluster correlation.*

1 $$ {n}_{opt}=\frac{C}{c_1+{c}_2{m}_{opt}\ } $$

2 $$ C={c}_1n+{c}_2 nm $$

3 $$ {m}_{opt}=\sqrt{\frac{\left(1-\rho \right){c}_1}{\rho {c}_2}} $$

where, *n*_*opt*_ is the number of required sample, *C* is the total cost of the survey, *c*_1_ is the unit cost per PSU for household lising and interview, *c*_2_ is the unit cost per individual interview, *n* is the total number of PSUs to be selected, *m* is the number of individuals to be selected in each PSU, and *ρ* is the intracluster correlation.

#### Data collection procedure

In this study, data from each country are nationally representative of each country’s eligible population. Eligible survey participants were surveyed through face-to-face interviews by a trained surveyor using the DHS model questionnaires. Data were collected by Measure DHS retrospectively using quantitative structural questionnaires which covered information on socio-demographic, reproductive health, access to services, and use of health services. Trained interviewers collected data via face-to-face interviews. All the data were collected at both household and individual levels of women still considered as reproductive (aged 15 to 49 years). The DHS dataset is publicly available; however, mailed consent was also taken as part of the Measure DHS protocol. Study participants were generated from the DHS as per the DHS protocol. Detailed information regarding survey sampling, quality control, management, and survey instruments are reported elsewhere [44]. Women were requested to provide information about CC screening knowledge along with awareness and utilisation of screening services. Written informed consent was taken from the respondents prior to conducting the survey. Rigorous data management was performed (e.g., data validity, reliability, quality control). This analysis considered the latest survey conducted by selected countries, and the data collection period was between 2008 and 2018. The survey response rate varied between 85 and 95%. The data set is publicly accessible after obtaining approval, which was received from the Measure DHS program.

A sample was drawn from the DHS database from each of the selected RCCs. After exclusion of non-responders and participants with missing data and unusual observations, data on 1,802,413 reproductive women living in these countries were included in the analysis (Table 1). India had the highest proportion of participants, followed by Burkina Faso and the Philippines. The average age ± Standard Deviation (SD) of the participants was 35.88 years (± 7.91 SD).

**Table 1:** Distribution of study population

Country	Type of survey	Survey years	Observation (N)
Albania	Standard DHS	2017–18	15,306
Bolivia	Standard DHS	2008	40,479
Burkina Faso	Standard DHS	2010	112,661
Colombia	Standard DHS	2015–16	11,804
Cote d’Ivoire	Standard DHS	2011–12	26,939
Dominican Republic	Standard DHS	2013	17,480
Egypt	Standard DHS	2014	9209
Equatorial Guinea	Standard DHS	2014–15	2561
Honduras	Standard DHS	2011–12	46,592
India	Standard DHS	2015–16	1,289,652
Jordan	Standard DHS	2012	40,386
Kenya	Standard DHS	2014	36,540
Lesotho	Standard DHS	2014	11,575
Namibia	Standard DHS	2013	16,953
Philippines	Standard DHS	2013	71,280
Tajikistan	Standard DHS	2012	20,449
Tanzania	Standard DHS	2011–12	10,869
Zimbabwe	Standard DHS	2015	21,677
Total		2008–2018	1,801,987

### Outcome variables

This study considered two outcome variables, namely ‘women’s knowledge and ‘utilisation of cervical cancer screening (CCS) services’. Participants were asked knowledge-specific questions related to CC screening services [50]. More specifically, questions such as ‘have you ever heard of a pap test’, ‘Do you know what a pap test is for?’, ‘Do you know what vaginal cytology is?’, ‘Have you ever heard of vaginal cytology?’, ‘How did you learn about vaginal cytology?’, ‘In the last 12 months, have you received educational information about cervical cancer screening?’ were asked to gather knowledge-related information on CC screening. The overall women’s knowledge surrounding CC screening services was measured as a dichotomous response (1 = ‘yes’ if the participant reported any positive response about CC screening services or 0 = ‘no’ otherwise). Further, participants were asked questions related to their CC screening service utilisation; for instance, questions associated with having a pap test, gynecologic examination or vaginal cytology examination, all of which depend on available services across countries [50]. Self-reported responses for CCS screening were considered and then categorised as ‘yes’ if the participant utilised any form of CCS or otherwise ‘no’ to measure the utilisation of CCS services.

### Explanatory variables

Explanatory variables were selected based on the socio-ecological model for the women’s knowledge and utilisation of CCS services [40, 41], and these data were examined for potential confounders [42]. Participants’ characteristics, which included age, education, sex of the household head and age at the time of respondent’s first childbirth, were considered as the predisposing factors in the analysis. Age was grouped as follows: < 26 years, 26–35 years, 36–45 years or ≥ 46 years. Educational background was defined as no education, primary education, secondary education or higher education. Household size was classified as < 5 members, 5–7 members, and more than 8 members. Media exposure was assessed by means of access to radio and/or television, whereas health insurance coverage and wealth status were considered mediator factors. Women’s history of breastfeeding, having amenorrhea, abstaining, currently working, access to mass media exposure and having health insurance coverage were dichotomous variables (‘yes’ if present or ‘no’ otherwise). Access to medical help for the self was categorised into three groups (1 = no problem, 2 = some problem, 3 = extreme problem). SES was based on the ownership of durable assets [40]. This method has been used in previous studies employing DHS data from developing countries [39, 41, 42]. Each household’s characteristics (assets) were dichotomised (‘yes’ if present and ‘no’ if not) [51]. Country-specific principal components analysis (PCA) was performed using ownership of durable assets [40]. Weights were estimated by factor scores derived from the first principal component in the PCA. The constructed wealth index values were then assigned to individuals based on accessible variables. The wealth index was divided into five strata: poorest (Q_1_: lowest 20%), poorer (Q_2_), middle (Q_3_), richer (Q_4_) and richest (Q_5_: top 20%) [52, 53]. Location of residence was dichotomised as either urban or rural [52, 53].

### Estimation strategies

#### Measuring and decomposing wealth-related inequalities

For the inequality analysis, comparisons of knowledge CC screening and utilisation of services were performed across wealth quintiles over the period specified. The standard measures of concentration index (Conc.I) were employed to examine the magnitude of household wealth-related inequality and the trends in CC screening knowledge and utilisation of services across 18 RCCs. The Conc. I was estimated as the covariance between knowledge and utilisation of CC screening services and the proportional rank in wealth score distribution [39] as follows:

$$ \mathrm{Conc}.\mathrm{I}=\frac{2}{n^2\overline{y}}\sum \limits_{i=1}^n{y}_i{r}_i\kern13.75em (1) $$

where Conc. I is the concentration index, $\overline{y}$ is the mean of knowledge and utilisation of CC screening services, r_i_ is the cumulative proportion that each individual represents over the total population once the distribution of wealth score has ranked the latter. The values of Conc. I are bounded between $\overline{y}-1$ and $1-\overline{y}$; $\overline{y}-1\le \mathrm{Conc}.\mathrm{I}\le 1-\overline{y}$ when y is dichotomous [41]. Conc. I acquires a negative value when the curve lies above the line of equality, which indicates a disproportionately lower prevalence of CC screening knowledge and utilisation of services among the poor (i.e., pro-poor). A positive value of Conc. I signifies a higher concentration of health indicators among the rich (i.e., pro-rich). There is no socioeconomic inequality in the distribution of CC screening knowledge and utilisation of services (y) when the value of Conc. I is zero and the concentration curve coincides with the 45° line. The dichotomous character of the knowledge and utilisation of CC screening services may result in unstable bounds in response to varying means; therefore, the normalised standard index was estimated to check the robustness of the estimation [42, 43]. In addition, when the outcome variable is dichotomous, the Conc. I has to be corrected in order to allow comparisons between groups of individuals from different time periods that may show different levels of use of health services [45]. In the context of a dichotomous outcome variable, the Erreygers’s Conc. I is the Conc. I multiplied by four times the mean health or outcome of interest [45]. Erreygers’ suggested corrected CI can be expressed as:

$$ E=\left(\frac{4\times \overline{y}}{y^{max}-{y}^{min}}\right)\ \mathrm{Conc}.\mathrm{I}\kern11em (2) $$

where *y*^*max*^ and *y*^*min*^ are the boundary of y (knowledge and utilisation of CC screening services). When the Erreygers’ corrected index is used, the decomposition of inequality is generally expressed as:

$$ E=4\times \sum \limits_k\left({\beta}_k^m{\overline{x}}_k\right) Conc.{I}_k+G{ConcI}_{\varepsilon}\kern5em (3) $$

This estimate produces an index that satisfies various attractive axiomatic properties for an inequality index, including the sign condition, scale invariance and mirror properties [46, 47]. The adjusted Conc. I method allows for an examination of the causes of (and their corresponding contributions to) and levels of changes in inequalities in terms of knowledge and utilisation of CC screening services [40]. In addition, multiple logistic regression was applied to measure the likelihood of CC screening knowledge, awareness and utilisation of services. Adjusted odds ratios (AORs) with a 95% confidence interval (CI) were estimated for identifying influencing factors on CC screening knowledge and utilisation of services at a 5% or lower level of significance. All the estimates were considered by sampling weights according to the DHS guideline. According to the DHS guideline, sample weights are estimated to six decimals but are presented in the standard recode files without the decimal point. They need to be divided by 1,000,000 before use to approximate the number of cases. As part of complex sample parameters when standard errors, confidence intervals or significance testing is required for the indicator [54]. All statistical analyses were performed with Stata/SE-13 software (StataCorp, College Station, TX, USA).

## Results

### Background characteristics of study participants

Table 2 features the background characteristics of the study participants. Nearly 75.31% of total participants belonged to the 26–45-year age group. Approximately 41.05% of all participants had no formal education, whereas 31.15% of participants had completed secondary education, followed by primary education (21.79%). A wide gap existed in who led the household, with nearly 85.17% of households being male–headed, and two–thirds of the women’s family consisting of five or more members. A sizeable number of women had no current exposure to breastfeeding (78.97%), amenorrhea (91.70%) and abstaining (93.23%). Approximately half of the women were employed, with 60.06% of women’s households having access to mass media communications. Further, 74.23% of participants had reported moderate or extreme problems in accessing medical care from health centres or other sources. Overall, just 20.25% of the households had health insurance coverage. Approximately 66.70% of households lived in a rural community, with roughly 44.44% of women having a low SES.

**Table 2:** Association of women’s knowledge of cervical cancer and utilisation of cervical cancer screening across participants characteristics

Characteristics	Number of observation	Women’s knowledge of cervical cancer			Utilisation of cervical cancer screening services		
	n (%)	n (%)	95% CI	*P*-value^1^	n (%)	95% CI	*P*-value^1^
Age group							
*< 26 years*	203,472 (11.75)	63,232 (31.08)	(30.88–31.28)		47,559 (26.02)	(25.82–26.22)	
*26–35 years*	629,486 (36.36)	217,696 (34.58)	(34.47–34.70)	< 0.001	175,494 (29.62)	(29.50–29.74)	< 0.001
*36–45 years*	674,297 (38.95)	241,054 (35.75)	(35.63–35.86)		199,553 (31.06)	(30.95–31.17)	
*46 or more*	223,878 (12.93)	79,957 (35.71)	(35.52–35.91)		67,803 (32.00)	(31.80–32.20)	
Educational level							
*no education*	752,174 (41.05)	203,970 (26.74)	(26.81–27.01)		148,425 (22.19)	(22.09–22.29)	
*primary*	399,246 (21.79)	140,437 (39.40)	(39.24–39.56)	< 0.001	116,728 (35.72)	(35.55–35.88)	< 0.001
*secondary*	570,867 (31.15)	208,976 (39.93)	(39.80–40.06)		182,532 (34.21)	(34.08–34.33)	
*higher*	110,019 (6.00)	45,628 (47.35)	(47.05–47.65)		42,724 (42.64)	(42.33–42.94)	
Household head							
*male*	1,527,282 (85.17)	558,890 (36.70)	(36.62–36.77)	< 0.001	414,864 (28.81)	(28.73–28.88)	< 0.001
*female*	265,922 (14.83)	104,376 (39.75)	(39.56–39.94)		79,670 (31.34)	(31.16–31.52)	
Household size							
*< 5 members*	534,170 (29.64)	189,376 (38.17)	(38.04–38.30)		16,4587 (32.61)	(32.48–32.74)	
*5–7 members*	858,427 (47.63)	318,362 (36.53)	(36.43–36.63)	< 0.001	239,802 (29.20)	(29.10–29.30)	< 0.001
*≥ 8 members*	409,815 (22.74)	156,481 (36.45)	(36.30–36.59)		90,147 (24.46)	(24.32–24.60)	
Currently breastfeeding							
*no*	1,341,835 (78.97)	463,050 (35.19)	(35.11–35.27)	< 0.001	403,083 (31.13)	(31.05–31.21)	< 0.001
*yes*	357,415 (21.03)	119,168 (31.63)	(31.47–31.78)		77,186 (23.82)	(23.67–23.96)	
Currently amenorrheic							
*no*	1,569,088 (91.70)	539,226 (34.69)	(34.61–34.76)		460,973 (30.57)	(30.49–30.64)	< 0.001
*yes*	141,967 (8.30)	51,881 (35.14)	(34.89–35.39)	0.312	29,434 (24.23)	(23.99–24.47)	
Currently abstaining							
*no*	1,595,179 (93.23)	553,764 (34.71)	(34.64–34.79)	< 0.001	465,436 (30.40)	(30.32–30.47)	< 0.001
*yes*	115,876 (6.77)	40,415 (34.88)	(34.60–35.15)		24,971 (25.40)	(25.13–25.67)	
Marital status							
*married*	1,529,410 (88.82)	517,451 (33.83)	(33.76–33.91)	< 0.001	408,194 (28.19)	(45.00–45.46)	< 0.001
*others*	192,514 (11.18)	83,854 (43.56)	(43.34–43.78)		82,213 (45.23)	(28.12–28.27)	
Employment status							
*no*	333,605 (51.07)	149,712 (44.88)	(44.71–45.05)	< 0.001	110,798 (36.11)	(35.94–36.28)	0.174
*yes*	319,666 (48.93)	165,495 (51.77)	(51.60–51.94)		93,590 (36.83)	(36.64–37.01)	
Access to health facility							
*no problem*	437,869 (25.77)	136,530 (31.18)	(31.04–31.32)		148,406 (32.60)	(32.46–32.73)	< 0.001
*some problem*	672,322 (39.57)	262,319 (39.02)	(38.90–39.13)	< 0.001	179,888 (29.45)	(29.33–29.56)	
*extreme problem*	588,996 (34.66)	186,316 (31.63)	(31.51–31.75)		151,967 (27.49)	(27.37–27.61)	
Health insurance coverage							
*no*	1,372,070 (79.75)	467,290 (34.06)	(33.98–34.14)	< 0.001	359,259 (27.86)	(27.78–27.94)	< 0.001
*yes*	348,436 (20.25)	147,864 (42.44)	(42.27–42.60)		114,053 (33.28)	(33.12–33.44)	
Mass media exposure							
*no*	704,804 (39.94)	214,934 (30.50)	(30.39–30.60)	< 0.001	120,414 (19.33)	(19.24–19.43)	< 0.001
*yes*	1,059,673 (60.06)	439,673 (41.49)	(41.40–41.59)		363,979 (35.13)	(35.04–35.23)	
Wealth Index							
*poorest (Q*_*1*_*)*	410,984 (22.92)	104,745 (25.49)	(25.35–25.62)	< 0.001	70,699 (18.76)	(18.64–18.89)	
*poorer (Q*_*2*_*)*	385,984 (21.52)	123,850 (32.09)	(31.94–32.23)		88,121 (24.39)	(24.25–24.53)	< 0.001
*middle (Q*_*3*_*)*	364,113 (20.31)	139,510 (38.32)	(38.16–38.47)		104,965 (30.52)	(30.36–30.67)	
*richer (Q*_*4*_*)*	341,052 (19.02)	149,721 (43.90)	(43.73–44.07)		114,233 (35.07)	(34.90–35.23)	
*richest (Q*_*5*_*)*	299,854 (16.64)	148,328 (48.68)	(44.52–55.29)		116,516 (40.19)	(35.16–42.92)	
Place of residence							
*urban*	600,094 (33.30)	271,061 (45.17)	(45.04–45.30)	< 0.001	206,979 (36.11)	(35.99–36.23)	< 0.001
*rural*	1,201,893 (66.70)	395,695 (32.92)	(32.84–33.01)		287,558 (25.65)	(25.56–25.73)	
Total	1,801,987 (100.00)	666,789 (36.99)	(36.92–37.06)		494,537 (29.19)	(29.12–29.25)	

^1^*P*-values were derived using chi-square test, *CI* confidence interval

**Table 3:** Comparison of the utilisation of CCS services between the present study findings (RCCs) and previous studies in HICs

Countries	Utilisation of CCS services, %		Degree of inequalities				Sources
	Poorest (Q_1_)	Richest (Q_5_)	RPD	RPR	(Q_5_-Q_1_) / Q_5_	Conc.I	
Present study findings (18 RCCs)	18.76	40.19	21.43	2.14	0.53	0.298	Present study
Austria	68.40	91.80	23.40	1.34	0.25	0.315	[36]
Australia	50.40	62.10	11.70	1.23	0.19	0.126	[55]
Brazil	55.40	88.40	33.00	1.60	0.37	0.296	[36]
Denmark	45.00	78.00	33.00	1.73	0.42	0.098	[56]
France	42.50	80.10	37.60	1.88	0.47	0.269	[36]
Finland	49.90	74.10	24.20	1.48	0.33	0.122	[36]
Germany	68.30	80.50	12.20	1.18	0.15	0.139	[36]
Greece	31.40	62.40	31.00	1.99	0.50	0.266	[36]
Hungary	38.60	78.30	39.70	2.03	0.51	0.285	[36]
Italy	72.10	69.90	−2.20	0.97	−0.03	0.061	[36]
Ireland	19.90	45.50	25.60	2.29	0.56	0.220	[36]
Luxembourg	79.20	88.90	9.70	1.12	0.11	0.243	[36]
Mexico	57.30	69.60	12.30	1.21	0.18	0.119	[36]
Netherlands	46.40	65.90	19.50	1.42	0.30	0.151	[36]
Paraguay	32.40	71.00	38.60	2.19	0.54	0.314	[36]
Portugal	18.70	71.30	52.60	3.81	0.74	0.318	[36]
Russia	60.50	77.00	16.50	1.27	0.21	0.142	[36]
Spain	44.00	72.30	28.30	1.64	0.39	0.207	[36]
Slovenia	62.50	78.40	15.90	1.25	0.20	0.273	[36]
Sweden	62.50	75.80	13.30	1.21	0.18	0.177	[36]
Slovakia	47.00	70.10	23.10	1.49	0.33	0.208	[36]
Uruguay	48.10	75.50	27.40	1.57	0.36	0.246	[36]
UK	56.80	63.40	6.60	1.12	0.10	0.069	[36]

Note: *CCS* cervical cancer screening, *RCCs* resource-constrained countries, *HICs* high-income countries, *Q*_*1*_ poorest socio-economic status, *Q*_*5*_ richest socio-economic status, *RPR* rich-poor ratio (Q_5_/Q_1_), *RPD* rich-poor difference (Q_5_-Q_1_), *Conc. I* Concentration Index

### Distribution of participants’ knowledge and utilisation of CCS services (for RQ 1)

**Fig. 2:**
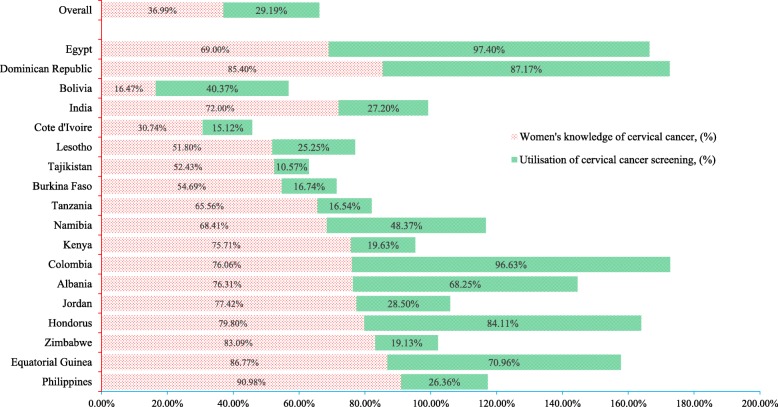
Distribution of women’s knowledge and utilisation of cervical cancer screening services across countries

**Fig. 3:**
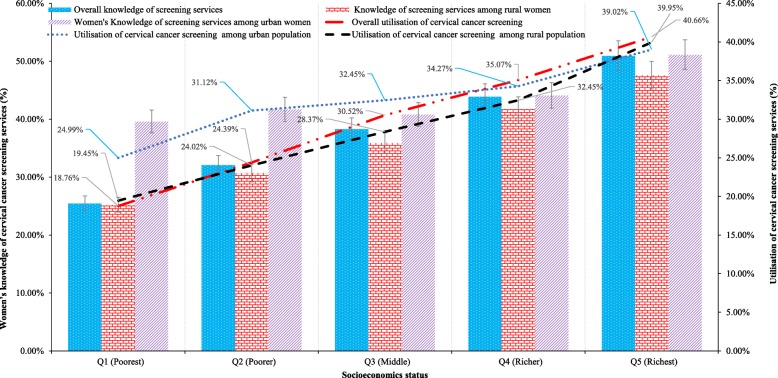
Unequal distribution of women’s knowledge surrounding cervical cancer (CC) screening services and utilisation of CC screening services by socioeconomic status

Approximately 37% of women had adequate knowledge about CCS services, with 29.19% of women having utilised the services, ranging from 10.57% in Tajikistan to 96.63% in Colombia (Fig. 2). The participants’ characteristics included in the analyses are predisposing factors (e.g., age, educational background, sex of household head, number of family members, marital status), enabling factors (e.g., employment status, access to medical help, health insurance coverage, access to mass media communications), and community and economic factors, all of which were significantly associated to women’s knowledge of screening services and utilisation (Table 2). Overall, women’s knowledge about screening services were significantly increased (*P* < 0.001) with a higher level of education (i.e., 26.74% for no formal education, 39.40% for primary school, 39.93% for secondary school and 47.35% for higher education). Female-headed households (39.75%) had slightly more knowledge about cervical cancer than male-headed ones (36.70%). Further, 42.44% of women’s households were insured, and 41.49% exposed to mass media had knowledge of CCS. Participants living in urban settings (45.17%) had more knowledge of screening services than rural women (32.92%). In addition, women’s level of knowledge and utilisation of CCS services were disproportionately greater for those of higher SES. For example, only 24.99% of the poorest women had knowledge of CC, whereas 39.95% of women in the richest strata did (Fig. 3). Similarly, 18.76% of the poorest women utilised CCS services, whereas 40.19% of women utilised in the richest quintile.

The magnitude of inequality in terms of utilisation of CCS services (rich-poor ratio, RPR = 2.14 times, concentration index, Conc. I = 0.298) were disproportionately concentrated among the most socioeconomic advantaged households (Table 3). A similar distribution was found across participants’ characteristics for utilising screening services in RCCs. Compared to the existing study’s findings that conducted in high income countries, the magnitude of inequality in terms of utilisation of CCS services were disproportionately also concentrated among women in the most socioeconomic advantaged households, including Austria (RPR = 1.34 times, Conc. I = 0.315), Australia (RPR = 1.23 times, Conc. I = 0.126), Brazil (RPR = 1.60 times, Conc. I = 0.296), France (RPR = 1.88, Conc. I = 0.269), Greece (RPR = 1.99 times, Conc. I = 0.269), Hungary (RPR = 2.03 times, Conc. I = 0.285), Ireland (RPR = 2.29 times, Conc. I = 0.220), Luxembourg (RPR = 1.12 times, Conc. I = 0.243) and UK (RPR = 1.12, Conc. I = 0.069).

### Factors influencing women’s knowledge and utilisation of CC screening services (for RQ 2)

Several factors influence women’s knowledge of screening and utilisation of CC screening services (Table 4). For example, age (OR = 1.03; 95% CI:1.01, 1.05; *P < 0.01*), year of schooling (OR = 1.08; 95% CI: 1.05, 1.11; *P < 0.001*), breastfeeding practices (OR = 1.03; 95% CI: 1.01, 1.05; *P < 0.05*), having amenorrhea (OR = 1.23; 95% CI: 1.19, 1.27; *P < 0.01*), employed women (OR = 1.39; 95% CI: 1.39, 1.41; *P < 0.01*), problems accessing medical help (OR = 1.55; 95% CI: 1.52, 1.58; *P < 0.01*), living in urban locations (OR = 1.13; 95% CI: 1.13, 1.15; *P < 0.01*) and higher wealth (OR = 1.26; 95% CI: 1.26, 1.27; *P < 0.001*) had a significant impact on possessing increased knowledge of CC compared with their counterparts. However, women from male-headed households (24.00%, OR = 0.76; 95% CI: 0.74, 0.77; *P < 0.05*) and having no mass media exposure (16.00%, OR = 0.84; 95% CI: 0.83, 0.85; *P < 0.001*) had low-level knowledge of screening services. Similarly, a number of factors significantly drove higher rates of utilisation of CCS services, including being married (OR = 2.11; 95% CI: 2.07, 2.15; *P < 0.05*), insured (OR = 1.58; 95% CI: 1.55, 1.61; *P < 0.01*) and being a woman in the richest households (OR = 2.00; 95% CI: 1.09, 2.11; *P < 0.01*). Participants’ with a current practice of abstaining (OR = 0.79; 95% CI: 0.76, 0.82; *P < 0.01*), not having access to mass media communications (OR = 0.43; 95% CI: 0.39, 0.47; *P < 0.01*) and living in a rural community (OR = 0.91; 95% CI: 0.89, 0.93; *P < 0.01*) were less likely to utilise CCSs.

**Table 4:** Inequality decompositions of the Erreygers’s concentration index for women’s knowledge of cervical cancer and utilisation of cervical cancer screening

Variables	^1^OR (95% CI)	Elast.	Erreygers’sConc.I	RC to the Erreygers’s Conc.I, % (95% CI)
Knowledge of cervical cancer				
Age (years)	1.03** (1.01, 1.05)	0.49	0.71	25.62*** (10.12, 30.59)
Schooling (years)	1.08* (1.05, 1.11)	0.32	0.36	15.07*** (10.26, 19.57)
Household head (= male)	0.76*** (0.74, 0.77)	− 0.24	0.01	−2.24*** (−3.10, −1.59)
Household size	0.99 (0.99, 1.00)	− 0.01	− 0.25	8.59 (− 2.36, 25.65)
Currently breastfeeding (= yes)	1.03*** (1.01, 1.05)	0.02	0.11	6.51 (− 0.25, 12.69)
Currently amenorrhea (= yes)	1.23*** (1.19, 1.27)	0.02	− 0.06	− 1.37*** (− 2.37, − 1.05)
Currently abstaining (= yes)	0.93** (0.90, 0.96)	− 0.01	− 0.03	0.32 (− 0.24, 2.38)
Marital status (= married)	1.02* (1.00, 1.04)	0.01	− 0.01	− 0.08 (− 1.21, 0.09)
Currently working status (= yes)	1.39*** (1.37, 1.41)	0.15	0.06	15.21*** (11.23, 59.45)
Access to health facility (= yes)	1.55*** (1.52, 1.58)	0.33	−0.24	− 10.00** (− 12.65, −4.89)
Health insurance coverage (= yes)	0.66*** (0.65, 0.67)	− 0.08	0.04	−6.94*** (− 9.58, − 4.29)
Mass media exposure (= no)	0.84*** (0.83, 0.85)	−0.15	− 0.26	15.26** (5.16, 23.36)
Place of residence (= urban)	1.13*** (1.11, 1.15)	0.09	−0.49	−9.76** (− 12.59, −5.69)
Wealth score	1.26*** (1.25, 1.27)	0.64	0.61	24.00* (3.68, 55.23)
Total				80.20** (60.55, 89.65)
Utilisation of cervical cancer screening				
Age (years)	1.03** (1.02, 1.05)	0.79	0.51	29.00*** (10.20, 39.51)
Schooling (years)	0.99* (0.98, 0.99)	−0.08	0.36	17.00** (12.59, 51.16)
Household head (= male)	1.12*** (1.10, 1.15)	0.12	0.01	3.33 (−2.36, 6.23)
Household size	0.98* (0.97, 0.98)	−0.11	−0.25	1.29 (−1.20, 2.31)
Currently breastfeeding (= yes)	1.16*** (1.14, 1.19)	−0.03	− 0.12	9.51*** (2.59, 12.59)
Currently amenorrhea (= yes)	0.96** (0.93, 0.99)	−0.01	− 0.06	0.63 (−2.16, 2.13)
Currently abstaining (= yes)	0.79*** (0.76, 0.82)	−0.01	− 0.03	2.04* (0.59, 4.57)
Marital status (= married)	2.11*** (2.07, 2.15)	0.09	−0.02	−8.23*** (−12.46, −5.80)
Currently working status (= no)	0.86*** (0.85, 0.88)	− 0.05	0.061	− 14.16*** (−19.23, − 8.47)
Access to health facility (= no)	0.73*** (0.72, 0.74)	−0.18	− 0.24	11.22* (2.36, 19.49)
Health insurance coverage (= yes)	1.58*** (1.55, 1.61)	0.07	0.04	8.08* (1.26, 9.45)
Mass media exposure (= no)	0.43*** (0.39, 0.47)	−0.70	−0.26	6.72** (1.56, 16.81)
Place of residence (= urban)	0.91** (0.89, 0.93)	0.09	−0.49	−9.76*** (−15.62, −6.85)
Wealth score	1.99*** (1.09, 2.11)	0.14	0.62	27.00** (14.56, 45.65)
Total				83.69*** (75.85, 95.54)

*OR* odds ratio, *CI* confidence interval, *Elast* elasticity, *Conc.I* concentration index, *RC* relative contribution, ^1^ORs were derived using logit regression model, ***P < 0.001, **P < 0.01, *P < 0.05

### Decomposition of women’s knowledge and utilisation of CCS services (for RQ 3)

The results of the analysis comprising the elasticity of odds of knowledge and utilisation of CC screening with respect to each factor are shown in Table 4. Higher elasticity values were determined for women’s age, year of schooling, sex of household head, employment status, access to medical assistance, mass media exposure, and wealth score determinants of women’s knowledge and utilisation of CCS services. The higher values of elasticity signified that these factors had a significant impact on women’s knowledge of screening as well as utilisation of screening services. Negative values for CI of the determining factors for both knowledge and utilisation of CC screening include women currently experiencing amenorrhea, abstaining, experiencing problems in accessing medical assistance and not having mass media exposure. These factors were significantly concentrated on economically disadvantaged households. In addition, this study also found that male-headed households (− 2.24%; 95% CI: − 3.10%, − 1.59%; *P < 0.01*), currently experiencing amenorrhea (− 1.37%; 95% CI: − 2.37%, − 1.05%; *P < 0.05*), having no problem accessing medical assistance (− 10.00%; 95% CI: − 12.65%, − 4.89%; *P < 0.05*), being insured (− 6.94%; 95% CI: − 9.58%, − 4.29%; *P < 0.01*) and place of residence (− 9.76%; 95% CI: − 12.59%, − 5.69%; *P < 0.05*) contributed to reducing inequality in women’s knowledge of screening services. Similarly, marital status (− 8.23%; 95% CI: − 12.46%, − 5.80%; *P < 0.01*), current work status (− 14.16%; 95% CI: − 19.23%, − 8.47%; *P < 0.05*) and place of residence (− 9.76%; 95% CI: − 15.62%, − 5.80%; *P < 0.01*) were observed to be significant factors that contributed to reducing inequality in the utilisation of CCS. However, factors that have the most positive contributions to the inequality in knowledge and utilisation of CC services include age (*P < 0.01)*, years of schooling (*P < 0.01)*, currently breastfeeding (*P < 0.01)*, mass media exposure (*P < 0.01)* and wealth score (*P < 0.01)*.

## Discussion

RCCs, over the past couple of decades, have witnessed remarkable progress in population health improvements by introducing a range of health-related interventions. However, socio-economic inequality is still a leading contributor to the lack of access to health promotion activities and utilisation of healthcare services. Our findings in this study suggested that socio-economic inequality was the most dominant predictor driving inequalities in women’s knowledge and utilisation of CCS services in RCCs. This study showed that knowledge of CCS and service utilisation is relatively poor among women, but that this knowledge did, however, vary widely across the relevant countries. The level of knowledge and utilisation of CCS were disproportionately distributed the higher proportion was in the richest wealth quintile at a level that was significantly higher than that of the poorest quintile. The analysis demonstrated a range of characteristics in the trends. Wealth score is one of the main factors that positively contributed to inequality levels in women’s knowledge of screening services. Factors that also contributed significantly to improvements include mass media exposure, current working status and schooling. On the contrary, factors that played a vital role in reducing inequality in women’s knowledge of CC included access to medical help, urban residence and health insurance coverage.

Women from disadvantaged socioeconomic households were found to have suboptimal knowledge of CC and underutilised CC screening services compared to their counterparts from more advantaged households, confirming findings reported by others [22, 23, 29, 36, 37]. Unfortunately, opportunistic CCS services are usually practised in RCCs [4, 21, 30, 57]. This method of provision of screening services is less effective because it primarily targets a small proportion of women who have the chance to come in contact with health care providers either in a health facility or within the community [31, 35, 57]. These opportunistic screening services are not widely available; where they are available, the service is grossly underutilised [20, 30, 31]. However, a wider variation was observed between awareness about CC, knowledge of the disease and utilisation of CC screening services [4, 20, 30, 31, 35, 39, 58, 59]. Community mobilisation, peer-to-peer engagement and organising health systems to track and follow-up with targeted women should play a substantial role part mitigating barriers and ensuring increased utilisation of screening services.

The decomposition analysis also revealed that the disparities in women’s knowledge of CC and utilisation of CCS were demonstrated by socio-economic inequalities via factors such as demographic characteristics (e.g., age, educational attainment, married), access to health facilities, currently breastfeeding, living in an urban community and mass media communications. Certain studies have shown that women living in deprived households with low levels of schooling and with a disproportionately lower level of knowledge experienced the lowest utilisation of screening services [22, 36]. These women have limited knowledge and practice in terms of use/non-use of sanitary pads, access to hygiene facilities, improper personal hygiene, health communication, health services and medical complications [4, 21, 60, 61]. Further, a number of factors were also highly related to these women such as a feeling of embarrassment, perceived pain during screening and unsupportive family members (e.g., husband, mother-in-law) owing to a lack of adequate knowledge of CC [62] and screening services [35, 60, 61]. Although differences in the assessment of knowledge about cancer make comparisons difficult, the level of knowledge about CCS is, however, suboptimal and of concern because women’s knowledge plays a primary role in increasing the rate of screening uptake [28, 63]. To facilitate acceptance of CC screening services, sensitisations should be carried out to increase awareness of the disease and the significance of screening [60]. Further, efforts should be focused on reducing identified barriers (such as fear of testing, outcome and consequences, financial constraints), strengthening health systems’ capacity and making use of female health workers to carry out screening services [60]. To diminish the existing inequalities in women’s knowledge and utilisation of CC screening in RCCs, establishment of the contributors of inequalities along with formulation and implementation of effective policies are necessary. Further, behavioural change through health promotion interventions could be an effective strategy for reducing the disparity of women’s knowledge and utilisation of screening services within poorer households and for women with low levels of schooling. In addition, community awareness and mass media communication (e.g., radios, newspapers) can also increase the utilisation rate of CC screening services [60].

There were several strengths of this study. Firstly, this research attempted to decompose wealth-related inequalities of both women’s knowledge and utilisation of CCS services by considering a number of countries. Secondly, the study reported determinants and decomposed wealth-related inequalities separately for both outcomes. Furthermore, the current study has analysed data on 1,802,413 reproductive women to obtain precise estimates. Hence, it is expected that the main findings of the work are likely to be similar for most other developing countries and therefore can assist policymakers in other nations.

Some limitations of this study also exist. All information collected from the reproductive age women was self-reported, representing an issue in terms of recall and social desirability biases. Recall bias and under-reporting of knowledge and utilisation of CC screening services may affect subsequently inferences. Further, the collected data may be less accurate than medical records that may have slight effect on the precision of the study outcome. Future studies might confirm these results using better quality data. Moreover, the data collection period was different for individual countries. To overcome this limitation, data were pooled from the DHS’ from multiple countries for the analysis, ignoring data collection time and country setting, an approach that may lead to biased estimates and weaken the generalisability of the study findings. Finally, the nature of the cross-sectional study does not allow for exploring the causal inference of knowledge and utilisation of CCS services [52, 53].

## Conclusions

In conclusion, the result of the present study revealed that a pro-rich inequality exists in women’s knowledge and utilisation of CCS services in RCCs. Wealth score, followed by mass media exposure, current working status and schooling explained a high proportion of inequality in women’s knowledge of screening services, whereas access to medical assistance, urban residence and health insurance coverage played a role in reducing these issues. As part of the present country-wise national priorities, initiatives should, therefore, be considered to address inequality and service utilisation. This aim will necessitate targeting underprivileged women with specific health interventions, with the object of ensuring accessibility to and affordability of adequate health care services in relation to the protection and promotion of women’s health. In addition, taking into account the societal perspective, policy efforts should be explored that mitigate structural factors, such as unequal wealth distribution, by focusing on effective financial mechanisms, social safety net programs and the creation of employment opportunities, all of which might contribute to reducing inequalities in health outcomes. Thus, to lower the socioeconomic inequalities that prevail in women’s knowledge and utilisation of CCS services in developing countries, interventions should be concentrated on these factors. In addition, an effective policy strategy should be developed through active collaboration among the different health systems along with the social and economic sectors to diminish wealth-related inequalities in women’s knowledge and utilisation of CCS services in low-resource settings.

## Data Availability

The DHS data are publicly accessible and were made available to us upon request by Measure DHS (https://dhsprogram.com/data/available-datasets.cfm).

## Abbreviations

RCCs: Resource-constrained countries
CC: Cervical cancer
CCS: Cervical cancer services
HPV: Human papillomavirus
SES: Socio-economic status
SEM: Socio-ecological model
DHS: Demographic and health survey
PCA: Principal components analysis
Conc.I: Concentration index
